# Low‑*Q* Asymptotic Behavior of the Effective Structure Factor Yields Model-Independent Radius of Interparticle Interaction (*R*_*i*_)

**DOI:** 10.1021/acsmeasuresciau.5c00099

**Published:** 2025-11-07

**Authors:** Chelsea E. R. Edwards, Wellington C. Leite, Yun Liu

**Affiliations:** † NIST Center for Neutron Research, 10833 National Institute of Standards and Technology, Gaithersburg, Maryland 20899, United States; ‡ Neutron Scattering Division, 6146 Oak Ridge National Laboratory, Oak Ridge, Tennessee 37830, United States; § Department of Chemical and Biomolecular Engineering, University of Delaware, Newark, Delaware 19716, United States

**Keywords:** particle interactions, radius of gyration, colloidal solutions, small-angle scattering, neutron scattering, Guinier analysis, structure factor

## Abstract

Guinier analysis has been extensively used in academic and industrial research settings to obtain the model-independent size of a polymer, protein, or colloid in solution from small-angle scattering data. Using the Guinier model, the radius of gyration (*R*_*g*_) is extracted from the form factor at low *Q*. Here, we develop an analogous approach for analyzing the effective structure factor data at low *Q* to extract a model-independent radius of interaction potential, *R*_*i*_. Whereas *R*_*g*_ describes how spread out the scattering length density distribution of particles is from their center of mass, *R*_*i*_ is an effective root-mean-square distance that quantifies how far the interparticle correlation deviates from its ideal gas configuration due to interactions. We demonstrate this novel analysis method by applying it to experimental small-angle neutron scattering data on lysozyme protein solutions. We discuss its broad implications for analysis of low-*Q* asymptotic X-ray and neutron scattering data, where Guinier analysis is traditionally applied.

## Graphical abstract

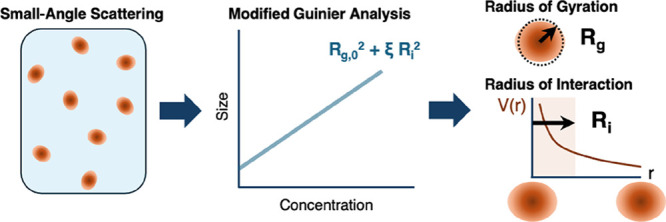

## 1 Introduction

Small-angle X-ray and neutron scattering (SAXS and SANS) methods are convenient and well-established techniques used to measure the relevant angstrom-to-micron length scales across biology and soft matter systems, elucidating structure–property relationships in materials like polymers, proteins, and micelles. The 1D scattering pattern, *I*(*Q*), for a solution of relatively monodisperse particles with number density, *n*, can be generally expressed as, *I*(*Q*) = *nP*(*Q*)*S*(*Q*),^1–3^ where *Q* is the magnitude of the scattering wave vector, **Q**; *P*(*Q*) is the (single-particle) form factor; and *S*(*Q*) the interparticle structure factor. Among many analysis models of scattering data, the Guinier model is among the most well-established,^4^ and is commonly used to obtain the radius of gyration of a typical particle in solution.^5,6^ Guinier analysis is applicable at low particle concentration or dilute conditions where interparticle interactions are minimal, so *S*(*Q*) = 1 is typically assumed and *I*(*Q*) ≈ *nP*(*Q*) = *n*|*F*(*Q*)|^2^. In the limit of small *Q*, the “shape function”, *F*(*Q*), which is the Fourier transform of the scattering length density (SLD) distribution ρ­(**r**), given by *F*(*Q*) = ∫ ρ­(**r**)*e* ^*i* **Q**·**r**^ *d* **r**, is approximated with a Taylor expansion of the phase factor *e* ^*i* **Q**·**r**^ to arrive at the Guinier law,

$$I\left(Q\right)\approx I\left(0\right)e^{-\frac{1}{3}Q^{2}{R_{g}}^{2}}$$ 1

Note that, because all orientations of particles in the solution are equally likely, we use scalar *Q* for *F*(*Q*). Eq 1 enables model-free determination of the radius of gyration, *R*_*g*_, directly from small-angle scattering data, given by

$${R_{g}}^{2}=\frac{\int \Delta \rho (r)r^{2}dr}{\int \Delta \rho (r)dr}$$ 2

Here, Δρ­(**r**) is the SLD difference between particles and solvent.

In Guinier analysis, *R*_*g*_^2^ is defined as the mean squared displacement (MSD) of the particle’s SLD distribution relative to the solvent SLD, i.e. *how spread out a particle is from its apparent center of SLD*. For homogeneous scattering objects, the value of *R*_*g*_ as measured by scattering is equivalent to the true value of *R*_*g*_, which reflects the mass density distribution about the center of mass of particles. By focusing on the low-*Q* asymptotic behavior of *P*(*Q*), the Guinier model allows us to obtain the size information of a particle without having *a priori* knowledge of the shape and morphology of particles.

*S*(*Q*) is determined by the relative positions of particles in solution, which has a strong impact to the low-*Q* region when *n* is large. For systems at equilibrium, *S*(*Q*) is a thermodynamic quantity determined by the interparticle potential, *V*(**r**). For monodisperse, isotropic particles with isotropic distribution,^7^

$$S(Q)=1+n\int h(r)e^{iQ\cdot r}dr$$ 3

where *h*(**r**) is the total correlation function. *h*(**r**) = *g*(**r**) – 1 represents the deviation of the local particle density from a uniform distribution, or more explicitly it is the difference in probability between a uniform distribution and the real system, where the probabilistic distribution is described by the pair distribution function, *g*(**r**). Therefore, measuring *I*(*Q*) of concentrated particle solutions has been widely used to probe particle–particle interactions, *V*(**r**), in solution.

Information about interparticle potential is traditionally obtained by fitting *I*(*Q*) data from concentrated samples, which requires modeling both *S*(*Q*) and *P*(*Q*) simultaneously. While it is typically straightforward to estimate *P*(*Q*) if the solute’s structure details are known or can be obtained from by measuring *I*(*Q*) in dilute solution, modeling *S*(*Q*) has proven to be challenging. For systems with isotropic interactions, if *V*(**r**) is known, *S*(*Q*) can be obtained by solving the Ornstein–Zernike (OZ) equation.^7–9^ Typically, this requires a closure relationship, e.g. Percus–Yevick, Hypernetted Chain, etc.,^8^ whose accuracy varies with the interaction potential. Analytically solving the OZ equation is very difficult, with solutions developed for only a few isotropic *V*(**r**).^10–14^ In recent years, numerical methods have been used to solve the OZ equation by fitting experimental data,^15,16^ and efforts to implement thermodynamic self-consistency have been developed to improve the accuracy of OZ equation solutions.^15,17–19^ However, these approaches are not yet widely used, and all are limited to isotropic *V*(**r**). For anisotropic interparticle interactions, evaluating *S*(*Q*) faces even steeper challenges. The Reference Interaction Site Model (RISM), which makes an additional approximation when solving the OZ equation, was developed several decades ago to address this issue^7,20^ and was extended to polymer systems.^21^ However, the broad applicability and widespread accuracy of these approaches across colloidal and protein systems are still not widely investigated.

To bypass the challenge of solving the OZ equation for anisotropic particles, Liu et al. recently introduced the generalized second osmotic virial coefficient, *B*_22_(*Q*), a function that encodes how net pairwise interactions vary across length scales and satisfies *S*(*Q*) = 1 – 2*nB*_22_(*Q*) under dilute conditions.^22^ It is an extension of the traditional measurement of the second virial coefficient *B*_22_ (or *B*_22_ *(Q=0*) ), which quantifies the interaction information averaged over distance and orientation between solute–solute pairs in a single parameter. *B*_22_ has been cataloged across numerous protein, polymer, and other colloidal systems, typically using static light scattering measurements,^23–25^ and has been linked to viscosity^26^ and the tendency of proteins to aggregate,^27^ among other applications.^28^ Whereas *B*_22_ is defined at *Q* = 0, this approach demonstrates its measurement at finite nonzero *Q*-values using SAXS or SANS. And the analytical theory to calculate *B*_22_(*Q*) or *S*(*Q*) at dilute conditions is developed for any isotropic or anisotropic interaction model and particle shape.^22^

Still, many experiments and simulations focus on the small *Q* limit due to the complexity in modeling *S*(*Q*) and *B*_22_(*Q*). Therefore, similar to the Guinier equation that describes low-*Q* form factor scattering data and returns a shape-independent size parameter (*R*_*g*_), it would be useful to have an equation that describes the asymptotic low-*Q* behavior of *S*(*Q*) and returns a shape-independent interaction parameter.

In this work, we develop a universal theory to model the *S*(*Q*) data at low *Q*. This theory can then be used to model *I*(*Q*) at low *Q* for any sample concentration using a method similar to the traditional Guinier analysis. Our theory introduces a new interaction length scale analogous to *R*_*g*_, which is extracted directly from the experimental small-angle scattering data. This new length scale, *R*_*i*_, defined as

$${R_{i}}^{2}=\frac{\int h\left(r\right)r^{2}dr}{\int h\left(r\right)dr}$$ 4

is the MSD of the interparticle correlation, i.e. *how far a typical particle deviates from the uniform distribution due to interparticle interactions*. For low-concentration systems with weak interactions, *R*_*i*_ is directly related to the interparticle potential, *V*(**r**), as ${R_{i}}^{2}\approx \frac{\int V(r)r^{2}dr}{\int V(r)dr}$ . We thus call this term the “radius of interaction potential” (or “interaction radius”). While in some fields *B*_22_^1/3^ could also be considered as a thermodynamic length scale related to pair interactions, since *B*_22_ is the system-averaged effective interaction volume of a particle pair, this length scale only reflects the net magnitude of interactions. Conversely, *R*_*i*_ reflects the *details of the shape of the interparticle potential* when providing a characteristic length scale of interparticle interactions. *R*_*i*_ can thus differentiate between systems with predominantly short-ranged versus long-ranged interparticle interactions, even if the *B*_22_ values are the same.

As a result, the scattering analysis method introduced herein enables for the first time the net spatial extent of the correlation function to be obtained directly from experimental data, using a new parameter that simulation experts can also straightforwardly compute. The approach also provides a quantitative measure of how the apparent experimental *R*_*g*_ is influenced by the particle concentration and interparticle interactions. Analogous to the traditional Guinier analysis on *P*(*Q*), our method obtains interaction information in a model-independent way, without *a priori* knowledge of the interparticle potential or *S*(*Q*).

We begin by providing a derivation for our model, including both isotropic and anisotropic particles, as well as noting the modification to the Zimm equation that follows from the derivation. We also demonstrate the application of the method to extract *R*_*i*_ and concentration-independent *R*_*g*_ from example experimental SANS data using a model protein system of lysozyme in histidine buffer. In addition to introducing the model and its implementation, our goal is to demonstrate the utility and easy application of our method to any colloidal system where Guinier analysis might traditionally be applied. Broadly, the work provides a more accurate description of the overall size of biomacromolecules, such as proteins, while also applicable to polymers, lipid-based systems, and other colloids.

## 2 Theory

The classical Guinier law gives $P\left(Q\right)\approx P\left(0\right)e^{-\frac{1}{3}Q^{2}{R_{g}}^{2}}$ , with *R*_*g*_ (eq 2) obtained by analysis of the low-*Q* asymptotic behavior of SAXS/SANS data. Herein, we focus on developing the asymptotic equation describing *S*(*Q*) at low *Q*, from which we introduce a new parameter, *R*_*i*_, as the root-mean-square distance of the interparticle interactions (eq 4). The key equations from this section are summarized in Table 1.

**Table 1:** Summary of Key Equations for Both Isotropic and Anisotropic Particles

	General Case	Dilute Limit
Classical Guinier law: *Low*-*Q asymptotic behavior of P*(*Q*)		$I\left(Q\right)\approx I\left(0\right)e^{-\frac{1}{3}Q^{2}{R_{g}}^{2}}$
		Already assumes *S*(*Q*) = 1
Radius of gyration (*R*_*g*_)	${R_{g}}^{2}=\frac{\int \Delta \rho (r)r^{2}dr}{\int \Delta \rho (r)dr}$	
Radius of interparticle interaction (*R*_*i*_)	${R_{i}}^{2}=\frac{\int h(r)r^{2}dr}{\int h(r)dr}$	${R_{i}}^{2}=\frac{\int \left(e^{-V\left(r\right)/k_{B}T}-1\right)r^{2}dr}{\int \left(e^{-V\left(r\right)/k_{B}T}-1\right)dr}$
		For weak interactions *V*(**r**), ${R_{i}}^{2}\approx \frac{\int V(r)r^{2}dr}{\int V(r)dr}$
Low-*Q* asymptotic behavior of *S*(*Q*)	$S\left(Q\right)\approx S\left(0\right)e^{-\frac{1}{3}\xi Q^{2}{R_{i}}^{2}}$ , where $\xi =\frac{1}{2}\frac{S\left(0\right)-1}{S\left(0\right)}$	$S\left(Q\right)\approx S\left(0\right)e^{\frac{1}{3}nB_{22}Q^{2}{R_{i}}^{2}}$
Modified Guinier law: L*ow*-*Q asymptotic behavior of* *I*(*Q*)	$I\left(Q\right)\approx I\left(0\right)e^{-\frac{1}{3}Q^{2}{R_{g,\mathrm{obs}}}^{2}}$ , where ${R_{g,\mathrm{obs}}}^{2}={R_{g,0}}^{2}+{{\xi R}_{i}}^{2}$	$I\left(Q\right)\approx I\left(0\right)e^{-\frac{1}{3}Q^{2}{R_{g,\mathrm{obs}}}^{2}}$ , where ${R_{g,\mathrm{obs}}}^{2}={R_{g,0}}^{2}-{nB}_{22}{R_{i}}^{2}$

### 2.1 Case 1: Spherical Solutes

For a system with monodisperse particles with isotropic density distribution and isotropic interactions, the structure factor is given by eq 3. At the limit of *Q* → 0, the phase factor can be approximated using a Taylor expansion as $e^{iQ\cdot r}\approx 1+iQ\cdot r-\frac{{\left(Q\cdot r\right)}^{2}}{2}$ , which leads to

$$S(Q)\approx 1+n\left(\int h(r)dr+iQ\cdot \int h(r)rdr-\frac{1}{2}\int h(r)(Q\cdot r{)}^{2}dr\right)$$ 5

Because *S*(*Q*) is isotropic, we can perform the angular average of **Q**. Eq 5 can then be simplified by observing that *i* **Q** ·∫ *h*(**r**)**r**d**r** = 0, since *g*(**r**) is isotropic, and therefore *h*(**r**)**r** is odd. In addition, ∫ *h*(**r**)­(**Q** ·**r**)^2^d**r** = *Q* ^2^ ∫ *h*(**r**)*r* ^2^ *cos* ^2^(θ)­d**r**, whose average value over the solid angle is $\frac{1}{3}Q^{2}\int h(r)r^{2}dr$ . As a result,

$$S(Q)\approx 1+n\int h(r)dr-\frac{n}{6}Q^{2}{R_{i}}^{2}\int h(r)dr$$ 6

where we have introduced the interaction radius, *R*_*i*_^2^, defined in eq 4. Next, we observe that *S*(0) = 1 + *n*∫ *h*(**r**)­d**r**, so we can write eq 6 equivalently as

$$S\left(Q\right)\approx S\left(0\right)\left[1-\frac{1}{6}\frac{S\left(0\right)-1}{S\left(0\right)}Q^{2}{R_{i}}^{2}\right]$$ 7

Eq 7 can be rewritten by recognizing that it is the expansion of the exponential function in the limit of small *Q* ^2^, giving

$$S\left(Q\right)\approx S\left(0\right)e^{-\frac{1}{6}\frac{S\left(0\right)-1}{S\left(0\right)}Q^{2}{R_{i}}^{2}}$$ 8

This expression describes the low-*Q* asymptotic behavior of the structure factor *S*(*Q*), analogous to the Guinier law for the form factor *P*(*Q*).

Since the scattering intensity is given by *I*(*Q*) = *nP*(*Q*)*S*(*Q*),^3^ we can combine the low-*Q* expansion of *S*(*Q*) (eq 7) with Guinier law (eq 1) to obtain a modified Guinier expression that accounts for structure-factor effects at finite concentration as follows:

$$I\left(Q\right)\approx nP\left(0\right)S\left(0\right)e^{-\frac{1}{3}Q^{2}{R_{g,\mathrm{obs}}}^{2}}\approx I\left(0\right)e^{-\frac{1}{3}Q^{2}{R_{g,\mathrm{obs}}}^{2}}$$ 9

This equation takes the exact same form as the classical Guinier law; here, *R*_*g*,obs_ is still the observed or apparent radius of gyration as measured by traditional Guinier analysis. However, by accounting for *S*(*Q*) in the derivation as shown herein, *R*_*g*,obs_ introduces two new parameters to more accurately describe the colloidal length scales,

$${R_{g,\mathrm{obs}}}^{2}={R_{g,0}}^{2}+\xi {R_{i}}^{2}$$ 10

where the constant $\xi =\frac{1}{2}\frac{S\left(0\right)-1}{S\left(0\right)}$ . In this equation, *R*_*i*_ is the radius of interparticle interaction introduced above, and *R*_*g*,0_ is the value of *R*_*g*,obs_ at the infinitely dilute limit (*n* = 0). In other words, *R*_*g*,0_ is the concentration-independent or “true” value of the radius of gyration as measured by small-angle scattering.

For a general or concentrated particle solution, *h*(**r**) is related with the potential of mean force, ω­(**r**), through $g\left(r\right)=e^{-\omega \left(r\right)/k_{B}T}$ , where *k*_*B*_ is the Boltzmann constant and *T* is the absolute temperature.

$${R_{i}}^{2}=\frac{\int (e^{-\omega (r)/k_{B}T}-1)r^{2}dr}{\int (e^{-\omega (r)/k_{B}T}-1)dr}$$ 11

#### 2.1.1 Dilute Limit

Further simplifications can be made in the dilute limit. At relatively dilute conditions, ω­(**r**) is the pairwise effective interaction potential, *V*(**r**). Under these conditions,

$${R_{i}}^{2}=\frac{\int (e^{-V(r)/k_{B}T}-1)r^{2}dr}{\int (e^{-V(r)/k_{B}T}-1)dr}$$ 12

If furthermore $\frac{V\left(r\right)}{k_{B}T}$ is small, i.e. the interaction potential is relatively weak, then the exponential term in eq 12 can be approximated with a Taylor series to give

$${R_{i}}^{2}\approx \frac{\int V(r)r^{2}dr}{\int V(r)dr}$$ 13

Under these conditions, *R*_*i*_^2^ is an approximate measure of the spatial extent or effective range of interparticle interaction as described by the potential *V*(*r*). In other words, for dilute solutions with weak potential, *R*_*i*_ *directly describes the typical distance over which the interaction potential is significant*.

Additionally in the dilute limit, ξ becomes $\frac{-nB_{22}}{1-2nB_{22}}$ , where *B*_22_ is the second virial coefficient, which at the limit of small *n* is ξ ≈−*nB*_22_. Therefore, eq 10 can be written as

$${R_{g,\mathrm{obs}}}^{2}\approx {R_{g,0}}^{2}-nB_{22}{R_{i}}^{2}$$ 14

In other words, the observed *R*_*g*_ from classical Guinier analysis varies linearly from its infinitely dilute value at a rate determined by interparticle interactions. Practically, the two parameters *R*_*g*,0_ and *R*_*i*_ can thus be extracted from a linear fit to *R*_*g*,obs_ data, provided that −*nB*_22_ can be calculated as shown in Section 4.

### 2.2 Case 2: Anisotropic Solutes

For anisotropic solutes with isotropic density distribution, we show here that the low-*Q* asymptotic behavior of the scattering intensity has the same final form as for spherical solutes, with

$$I\left(Q\right)\approx I\left(0\right)e^{-\frac{1}{3}Q^{2}{R_{g,\mathrm{obs}}^{A}}^{2}}$$ 15

where “A” simply denotes the anisotropic case. Again, ${R_{g,\mathrm{obs}}^{A}}^{2}$ can be also linked to the interaction radius by the following equation

$${R_{g,\mathrm{obs}}^{A}}^{2}={R_{g,0}}^{2}+\xi^{\prime }{\left({R_{i}}^{2}\right)}^{\prime }$$ 16

Here, the *′* denotes orientation-dependent averages of the interparticle correlation functions. Otherwise, the anisotropic modified Guinier equation and the dependence of *R*_*g*, obs_^*A*^ on the infinitely dilute *R*_*g*_ and radius of interparticle correlation match eqs 9 and 10 exactly. Thus, from an experimental perspective, the application and interpretation of the modified Guinier equation for anisotropic and spherical solutes is identical.

We derive eqs 15 and 16 as follows. For monodisperse anisotropic particles, the SANS/SAXS pattern can generally be expressed as

$$I\left(Q\right)=n{\left\langle P\left(Q,\Omega \right)\right\rangle }_{\Omega }S\left(Q\right)$$ 17

where ⟨*P*(**Q**, Ω)⟩_Ω_ indicates the average of the orientation-dependent form factor *P*(**Q**, Ω) over all particle orientations Ω, and *S*(*Q*) is the effective structure factor.^3,22^ Here and in the following we use Ω to refer to the orientation of a particle in 3D space; Ω_*i*_ refers to the orientation of particle *i*.

Liut et al. have shown recently that *S*(*Q*) in eq 17 is given by^22^

$$S\left(Q\right)=1+\frac{n}{{\left\langle P\left(Q,\Omega \right)\right\rangle }_{\Omega }}\langle \int dr{\left\langle F_{1}\left(Q,\Omega_{1}\right)F_{2}^{*}\left(Q,\Omega_{2}\right)h\left(r,\Omega_{1},\Omega_{2}\right)\right\rangle }_{\Omega_{1},\Omega_{2}}e^{iQ\cdot r}\rangle_{\Omega_{1},\Omega_{2}}$$ 18

where the orientation-dependent shape function, *F*(**Q**, Ω) = ∫ ρ(**r**, Ω)*e* ^*i* **Q**·**r**^ *d* **r**, is defined in terms of ρ­(**r**, Ω), the SLD distribution function of solutes with orientation Ω. (We replace Δρ­(**r**) with ρ­(**r**) to simplify the equations in this section.) Thus, *F*_1_ and complex conjugate *F*_2_^*^ describe particles with orientation Ω_1_ and Ω_2_, respectively. The orientation-dependent total correlation function *h*(**r**, Ω_1_, Ω_2_) is given by *h*(**r**, Ω_1_, Ω_2_) = *g*(**r**, Ω_1_, Ω_2_) – 1. Whereas *g*(**r**, Ω_1_, Ω_2_) describes the probability of finding a particle at position **r** with orientation Ω_2_ given that there is a particle at the origin with orientation Ω_1_, *h*(**r**, Ω_1_, Ω_2_) describes the excess (*h* > 0) or deficit (*h* < 0) in that probability due to interactions, relative to a uniform distribution at the same density. In particular, $g\left(r,\Omega_{1},\Omega_{2}\right)=\frac{n\left(r,\Omega_{1},\Omega_{2}\right)}{n}$ , where *n*(**r**, Ω_1_, Ω_2_) is the conditional number density of particles with separation **r** and orientation Ω_1_, Ω_2_; and *n* is still the bulk (positionally and orientationally averaged) number density.

For randomly oriented particles in solution, averaging over **Q**-orientation does not affect *S*(*Q*) = ⟨*S*(*Q*)⟩_*Q*_, which is described by the expression

$$1+\frac{n}{\langle P(Q,\Omega )\rangle_{\Omega ,Q}}\langle \int dr\langle F_{1}(Q,\Omega_{1})F_{2}^{*}(Q,\Omega_{2})h(r,\Omega_{1},\Omega_{2})\rangle_{\Omega_{1},\Omega_{2}}e^{iQ\cdot r}\rangle_{\Omega_{1},\Omega_{2},Q}$$ 19

This simplifies to

$$S(Q)=1+\frac{n}{\langle P(Q,\Omega )\rangle_{\Omega ,Q}}\int dr\langle F_{1}(Q,\Omega_{1})F_{2}^{*}(Q,\Omega_{2})h(r,\Omega_{1},\Omega_{2})e^{iQ\cdot r}\rangle_{\Omega_{1},\Omega_{2},Q}$$ 20

As *Q* → 0, the phase factor in *F*_*i*_(**Q**, Ω_*i*_) can be approximated using a Taylor expansion as in eq 5, i.e.,

$$F_{i}\left(Q,\Omega_{i}\right)\approx \int \rho \left(r_{i},\Omega_{i}\right)\left(1+iQ\cdot r_{i}-\frac{1}{2}{\left(Q\cdot r_{i}\right)}^{2}\right)dr_{i}$$ 21

where **r**_*i*_ indicates distance to particles of orientation Ω_*i*_. This expression can be simplified analogously to eq 5 by observing that *i* **Q** ·∫ ρ­(**r**, Ω)**r** *d* **r** = 0 as we can define the center of the scattering length to be the origin. So,

$$F_{i}(Q,\Omega_{i})\approx \int \rho (r_{i},\Omega_{i})\left(1-\frac{1}{2}(Q\cdot r_{i}{)}^{2}\right)dr_{i}$$ 22

Therefore, the averaged integral in eq 20 becomes

$$\approx \int dr\langle \int dr_{1}\rho (r_{1},\Omega_{1})\left(1-\frac{1}{2}(Q\cdot r_{1}{)}^{2}\right)\int dr_{2}\rho (r_{2},\Omega_{2})\left(1-\frac{1}{2}(Q\cdot r_{2}{)}^{2}\right)h(r,\Omega_{1},\Omega_{2})e^{iQ\cdot r}\rangle_{\Omega_{1},\Omega_{2},Q}$$ 23

which expression can be further simplified to

$$\approx \int dr\int dr_{1}\int dr_{2}\langle \rho (r_{1},\Omega_{1}(\rho (r_{2},\Omega_{2})\left(1-\frac{1}{2}(Q\cdot r_{1}{)}^{2}-\frac{1}{2}(Q\cdot r_{2}{)}^{2}\right)h(r,\Omega_{1},\Omega_{2})e^{iQ\cdot r}\rangle_{\Omega_{1},\Omega_{2},Q}$$ 24

After Taylor expansion of the phase factor *e* ^*i* **Q**·**r**^ and only keeping the terms up to *Q* ^2^, this expression is equivalently

$$\approx \int dr\int dr_{1}\int dr_{2}\langle \rho (r_{1},\Omega_{1})\rho (r_{2},\Omega_{2})h(r,\Omega_{1},\Omega_{2})\left(1-\frac{1}{2}(Q\cdot r_{1}{)}^{2}-\frac{1}{2}(Q\cdot r_{2}{)}^{2}+iQ\cdot r-\frac{1}{2}(Q\cdot r{)}^{2}\right)\rangle_{\Omega_{1},\Omega_{2},Q}$$ 25

The *Q*-average can be distributed as

$$\approx \int dr\int dr_{1}\int dr_{2}\langle \rho (r_{1},\Omega_{1})\rho (r_{2},\Omega_{2})h(r,\Omega_{1},\Omega_{2})\left(1-\frac{1}{2}\langle (Q\cdot r_{1}{)}^{2}\rangle_{Q}-\frac{1}{2}\langle (Q\cdot r_{2}{)}^{2}\rangle_{Q}-\frac{1}{2}\langle (Q\cdot r{)}^{2}\rangle_{Q}\right)\rangle_{\Omega_{1},\Omega_{2}}$$ 26

which expression can be further simplified using the isotropic average ⟨(**Q** ·**r**)^2^⟩_*Q*_ = *Q* ^2^ *r* ^2^/3 to obtain

$$\approx \int dr\int dr_{1}\int dr_{2}\langle \rho (r_{1},\Omega_{1})\rho (r_{2},\Omega_{2})h(r,\Omega_{1},\Omega_{2})\left(1-\frac{1}{6}Q^{2}{r_{1}}^{2}-\frac{1}{6}Q^{2}{r_{2}}^{2}-\frac{1}{6}Q^{2}r^{2}\right)\rangle_{\Omega_{1},\Omega_{2}}$$ 27

Since *F*(*Q* = 0) is a constant, i.e. ∫ ρ­(**r**_*i*_, Ω_*i*_)­d**r**_*i*_ takes the same value for all particle orientations Ω_*i*_, we can define Λ ≡∫ ρ­(**r**_1_, Ω_1_)­d**r**_1_ = ∫ ρ(**r**_2_, Ω_2_)d**r**_2_. Note that *R*_*g*_ can be written equivalently as ${R_{g}}^{2}=\frac{\int \rho (r_{i},\Omega_{i}){r_{i}}^{2}dr_{i}}{\Lambda }$ (see eq 2). Plugging in gives

$$\approx \int dr\langle \Lambda^{2}\;h(r,\Omega_{1},\Omega_{2})\left(1-\frac{1}{3}Q^{2}{R_{g}}^{2}-\frac{1}{6}Q^{2}r^{2}\right)\rangle_{\Omega_{1},\Omega_{2}}$$ 28

To further simplify eq 20, the *Q*- and orientationally averaged form factor, ⟨*P*(**Q**, Ω)⟩_Ω,*Q*_ must also be evaluated with

$$\langle P(Q,\Omega )\rangle_{\Omega ,Q}\approx \langle P(0,\Omega )\rangle_{\Omega }\langle e^{-\frac{1}{3}Q^{2}{R_{g}}^{2}}\rangle_{Q}$$ 29

Note that *P*(0) = |*F*(0)|^2^ = Λ^2^. In addition, $\langle e^{-\frac{1}{3}Q^{2}{R_{g}}^{2}}\rangle_{Q}$ at a specific *Q*-value is simply $e^{-\frac{1}{3}Q^{2}{R_{g}}^{2}}\approx 1-\frac{1}{3}Q^{2}{R_{g}}^{2}$ , where the approximation from the Taylor expansion of the exponential is the same as that in the Guinier's law derivation. Therefore, we can rewrite eq 29 as

$${\left\langle P\left(Q,\Omega \right)\right\rangle }_{\Omega ,Q}\approx \Lambda^{2}\left(1-\frac{1}{3}Q^{2}{R_{g}}^{2}\right)$$ 30

Next, we plug the simplified expressions for the integral (eq 28) and the averaged form factor (eq 30) into the expression for the effective structure factor *S*(*Q*) for a solution of anisotropic particles with isotropic density (eq 20):

$$S(Q)\approx 1+\frac{n}{\Lambda^{2}(1-\frac{1}{3}Q^{2}{R_{g}}^{2})}\int dr\langle \Lambda^{2}h(r,\Omega_{1},\Omega_{2})\left(1-\frac{1}{3}Q^{2}{R_{g}}^{2}-\frac{1}{6}Q^{2}r^{2}\right)\rangle_{\Omega_{1},\Omega_{2}}$$ 31

which simplifies to

$$S(Q)\approx 1+n\int dr\langle h(r,\Omega_{1},\Omega_{2})\left(\frac{1-\frac{1}{3}Q^{2}{R_{g}}^{2}-\frac{1}{6}Q^{2}r^{2}}{1-\frac{1}{3}Q^{2}{R_{g}}^{2}}\right)\rangle_{\Omega_{1},\Omega_{2}}$$ 32

The fraction in the above equation can be further simplified as

$$S(Q)\approx 1+n\int dr\langle h(r,\Omega_{1},\Omega_{2})\left(1-\frac{1}{6}Q^{2}r^{2}\right)\rangle_{\Omega_{1},\Omega_{2}}$$ 33

which is equivalent to

$$S\left(Q\right)\approx S\left(0\right)-\frac{n}{6}Q^{2}\int dr\;r^{2}\langle h\left(r,\Omega_{1},\Omega_{2}\right)\rangle_{\Omega_{1},\Omega_{2}}$$ 34

Defining $h\left(\overset{̿}{r}\right)$ = ⟨*h*(**r**, Ω_1_, Ω_2_)⟩_Ω_1_,Ω_2__, where the double-overline denotes the average over the orientations of particles 1 and 2, then we may define $\overset{̿}{S\left(0\right)}=1+n\int \overset{̿}{h(r)}dr$ . Similarly, we define ${\left({R_{i}}^{2}\right)}^{\prime }=\frac{\int r^{2}\overset{®}{\overset{®}{h\left(r\right)}}dr}{\int \overset{®}{\overset{®}{h\left(r\right)}}dr}$ , which is essentially the same as eq 4. Using these definitions, we rewrite the effective structure factor as

$$S(Q)\approx \overset{̿}{S(0)}\left[1-\frac{1}{6}\frac{\overset{̿}{S(0)}-1}{\overset{̿}{S(0)}}Q^{2}({R_{i}}^{2}{)}^{\prime }\right]$$ 35

In the limit of small *Q* ^2^, this expression can be approximated by an exponential as

$$S(Q)\approx \overset{̿}{S(0)}e^{-\frac{1}{6}\frac{\overset{̿}{S(0)}-1}{\overset{̿}{S(0)}}Q^{2}({R_{i}}^{2}{)}^{\prime }}$$ 36

similarly to the spherical particle case (eq 8). As in the derivation for spherical solutes, we combine the above expression for *S*(*Q*) with the classical Guinier law to obtain the structure-factor-corrected Guinier expression for anisotropic solutes:

$$I(Q)\approx nP(0)\overset{̿}{S(0)}e^{-\frac{1}{3}Q^{2}{R_{g,\mathrm{obs}}^{A}}^{2}}$$ 37

which leads to eq 15. Note that this modified Guinier law is exactly the same as that for spherical particles (eq 9). Since the measured *I*(*Q*) will not differentiate between $\overset{̿}{S(0)}$ and *S*(0), the final equations for the anisotropic and spherical cases are identical from an experimental perspective.

### 2.3 Implications for Zimm Analysis

Given the close relationship between the Zimm equation and the Guinier law, we show here the modified form of the Zimm equation due to the structure factor effects that follows directly from the above equations. To derive the Zimm equation, the Guinier law (eq 1) can be rewritten as

$$\frac{n}{I(Q)}\approx P(0{)}^{-1}S(0{)}^{-1}e^{\frac{1}{3}Q^{2}{R_{g}}^{2}}$$ 38

The Zimm equation assumes dilute conditions and is typically written in terms of the second Virial coefficient *B*_22_(*Q* = 0) or *B*_22_, i.e., using $S{\left(0\right)}^{-1}=\frac{1}{1-2nB_{22}}\approx 1+2nB_{22}$ for *n* → 0. Thus, eq 38 can be approximated with a Taylor expansion to arrive at the equation:

$$\frac{n}{I\left(Q\right)}\approx \frac{1+2nB_{22}}{P\left(0\right)}\left(1+\frac{1}{3}Q^{2}{R_{g}}^{2}\right)$$ 39

Note that Zimm’s original 1948 equation,^29^ and also the form used in Lodge and Hiemenz,^30^ can be simply derived from eq 39 (see ESI Section 1).

To derive the modified Zimm equation, we follow this same procedure using our modified Guinier equation (eq 9). In reciprocal form, it is

$$\frac{n}{I(Q)}\approx P(0{)}^{-1}S(0{)}^{-1}e^{\frac{1}{3}Q^{2}{R_{g,0}}^{2}}e^{\frac{1}{3}Q^{2}\xi {R_{i}}^{2}}$$ 40

Again, *S*(0)^−1^ and the exponential terms can be approximated using Taylor expansion. We also plug in ξ ≈ −*nB*_22_. The resulting extension of the Zimm equation, which is relevant as *Q*, *n* → 0 is given by

$$\frac{n}{I\left(Q\right)}\approx \frac{1+2nB_{22}}{P\left(0\right)}\left(1+\frac{1}{3}Q^{2}{R_{g,0}}^{2}\right)\left(1-\frac{1}{3}nB_{22}Q^{2}{R_{i}}^{2}\right)$$ 41

As compared to eq 39, this modified Zimm equation has one additional term related to the interaction radius that also varies with the concentration *n*.

### 2.4 Expected Magnitude of *R*_*i*_ at Dilute Conditions

How far might we expect the particle configuration to deviate from the ideal gas configuration at relatively dilute conditions? As a first approximation, consider the hard sphere system, wherein spheres of uniform internal density and diameter σ = 2*R*_HS_, where *R*_HS_ is the radius, interact according to the classical hard sphere potential

$$u\left(r\right)=\{\begin{array}{ll} \infty , & r<\sigma \\ 0, & r\geq \sigma \end{array}$$ 42

In this case, the true hard-sphere radius of gyration is given by $R_{g,\mathrm{HS}}=\sqrt{\frac{3}{5}}R_{HS}=\frac{1}{2}\sqrt{\frac{3}{5}}\sigma$ . The radius of interparticle interaction is given by $R_{i,\mathrm{HS}}=\frac{\int (e^{-\beta u(r)}-1)r^{2}dr}{\int (e^{-\beta u(r)}-1)dr}$ , where $\beta =\frac{1}{kT}$ , which evaluates to $R_{i,\mathrm{HS}}=\sqrt{\frac{3}{5}}\sigma$ . Therefore, in this limiting case, we find that

$$R_{i,\mathrm{HS}}=2R_{g,\mathrm{HS}}$$ 43

or *R*_*i*_^2^ = 4*R*_*g*_^2^. Though this exact equivalence is unlikely to hold in a real system, we may expect similar orders of magnitude for *R*_*g*_ and *R*_*i*_. For systems involving significantly nonideal characteristics, like strongly attractive or sticky interactions and anisotropic or floppy solutes, determining the typical magnitude of *R*_*i*_ will require substantial future experimental characterization across a variety of real systems.

## 3 Materials and Methods

### 3.1 Materials

Lysozyme from chicken egg white was obtained as an off-white crystalline powder from MP Biomedicals (<3.5% chloride and activity >23,500 u/mg solid). Sodium chloride (NaCl) was obtained from Sigma-Aldrich (BioXtra: ≥99.5%). l-Histidine (98+%) and l-histidine hydrochloride monohydrate (98%) were obtained from Thermo Scientific Chemicals. Deuterium oxide (99.9% D_2_O) was obtained from Cambridge Isotope Laboratories, Inc.

### 3.2 Solution Preparation

Histidine buffer was prepared by dissolving 0.3870 g of l-histidine with 0.5242 g of l-histidine hydrochloride monohydrate in 200 mL of D_2_O using a volumetric flask. The buffer pH was measured to be 6.05. Next, NaCl was added to the solution by dissolving 0.8767 g of NaCl in 100 mL of the l-histidine buffer using a volumetric flask. The resulting final buffer consisted of 150 mmol/L NaCl in 25 mmol/L histidine buffer, pH 6 in D_2_O. The buffer was vacuum filtered with a Thermo Fisher Scientific Nalgene RapidFlow 50 mm filter unit.

To prepare the protein solutions, frozen crystalline lysozyme was brought to room temperature, massed on a microbalance (120.0 mg), and 6 mL of buffer was added to reach a nominal concentration of 20 mg/mL. This solution was mixed on a rocker for 30 min at room temperature. A concentration series was prepared by successive dilution of this initial stock solution to obtain nominal concentrations of 15, 10, 7.5, 5, and 2.5 mg/mL lysozyme. The buffer and protein solutions were prepared 4 days prior to the first day of the neutron scattering experiment, and were stored in a closed container away from light and sealed with parafilm, either at 4°C or on ice during transportation to the beamline.

### 3.3 Concentration and Volume Fraction

The exact mass concentration of lysozyme corresponding to each nominal concentration was measured using the NanoDrop One instrument (Thermoscientific) within 12 h of conducting the corresponding neutron measurement. The default lysozyme measurement settings were used: $\frac{\epsilon }{1000}$ = 26.4 at 280 nm for a 1% mass fraction lysozyme solution (10 mg/mL). At least 6 measurements were conducted per solution (2 μL each). The resulting average, median, and standard deviation for each concentration are shown in Table 2.

**Table 2:** Measured Lysozyme Concentrations [mg/mL], Number Density in Units [1/mL] ∗ 10^17^, and Volume Percent

Nominal Concentration	2.5	5	7.5	10	15	20
Median	2.46	4.92	7.40	9.95	14.14	18.59
Mean (*c*)	2.45	4.91	7.40	9.94	14.21	18.56
Standard Deviation of *c*	0.03	0.04	0.06	0.05	0.20	0.12
Number Density (*n*)	1.030	2.068	3.117	4.187	5.985	7.818
Standard Deviation of *n*	0.013	0.017	0.026	0.021	0.083	0.052
Volume % (100ϕ)	0.168	0.336	0.507	0.681	0.974	1.272
Standard Deviation of 100ϕ	0.002	0.003	0.004	0.003	0.014	0.009

Number densities *n* of lysozyme were calculated using the measured mean mass concentrations, *c*, from the nanodrop measurements using *M*_*W*_ = 14.3 kDa, its theoretical mass based on the amino acid sequence, and Avogadro’s number. Volume fractions ϕ were calculated as $\phi =\frac{c}{\rho }$ , where ρ is the mass density of the lysozyme protein. Though the spatial average of protein density is typically assumed to equal 1.35 g/cm^3^, it is actually molecular-weight-dependent, with smaller molecular-weight proteins like lysozyme having increased density. Using the empirical equation proposed by Fischer et al.,^31^ we find that ρ ≈ 1.46 g/cm^3^ for lysozyme protein, from which we calculate that the ϕ used herein ranges from 0.0017 to 0.013. See Table 2 for complete results for *n*, *c*, and ϕ for each nominal concentration.

### 3.4 Small Angle Neutron Scattering

Small-angle neutron scattering (SANS) experiments were performed at the Bio-SANS beamline (CG-3)^32^ of the high flux isotope reactor at Oak Ridge National Laboratory (ORNL). Samples were measured in 5 mm quartz cylindrical cuvettes (Hellma), and the sample temperature was maintained at 25°C using a Peltier device. To minimize error due to variations in path length between individual cuvettes, the exact same cuvette was used for both buffer and the sample measurements. SANS patterns were obtained in 60 minutes to provide sufficient signal-to-noise ratio.

SANS data were collected using a three-detector array. The small-angle detector was positioned at 7.0 m from the sample, the mid-range detector at 4.0 m with a 2.7° anticlockwise rotation relative to the direct beam, and the high-angle (curved wing) detector at 1.13 m with a 7.25° rotation from the direct beam. This configuration covered a momentum transfer range of 0.007Å^–1^ < *Q* < 0.85Å^–1^, where the magnitude of the scattering vector $Q=\frac{4\pi }{\lambda }$ sin $\left(\frac{\theta }{2}\right)$ with λ the wavelength and θ the scattering angle, using neutrons with a wavelength of 6.44 Å and a relative wavelength spread (Δλ/λ) of 0.123. The Panel Scan feature^33^ of the data acquisition system was used to control the instrument during measurement.

Data reduction was performed using the facility’s data reduction toolkit for small-angle neutron scattering (drt-SANS) software.^32^ The reduction included corrections for instrument background, detector sensitivity, and instrument geometry, followed by circular averaging to obtain 1D scattering profiles. Reference measurements of empty beam, beam center, and buffer backgrounds were measured and applied during data reduction with drt-SANS.

SANS data analysis was conducted on the resulting 1D profiles in scattering intensity. The average *I*(*Q*)-value between 0.35–0.45 Å was subtracted as background using a custom Python code. The background-subtracted *I*(*Q*) data were fitted using Guinier analysis as described below. Background intensity values and SANS data prior to background subtraction are shown in Figure S1.

## 4 Results

As shown in Section 2, the observed radius of gyration *R*_*g*,obs_ from traditional Guinier analysis is dependent on the concentration according to the interparticle interaction potential. In particular, *R*_*g*,obs_^2^ varies linearly from its infinitely dilute value—denoted by *R*_*g*,0_^2^ herein, which can also be thought of as a “true” *R*_*g*_-value—with a slope that depends on *R*_*i*_^2^, the squared “radius of interparticle interaction” that reflects the interactions between particles in the solution. This dependence is given by eq 14 and holds for both spherical and anisotropic solutes. In this section, we demonstrate the extraction of the parameters *R*_*g*,0_ and *R*_*i*_ from experimental small-angle neutron scattering (SANS) data on a model system of lysozyme protein in solution. Buffer conditions, sample preparation, and instrument configuration details are provided in Section 3.

SANS data for the tested lysozyme solutions after background subtraction are shown in Figure 1A as a function of nominal concentration. Data are shown after normalization by the protein volume fraction ϕ, calculated from the measured concentration (exact values provided in Table 2) in Figure 1B. In general, the normalized data overlay well, reflecting the accuracy of the concentration measurements and that there is only minimal effect of the structure factor on the scattering pattern.

**Figure 1:**
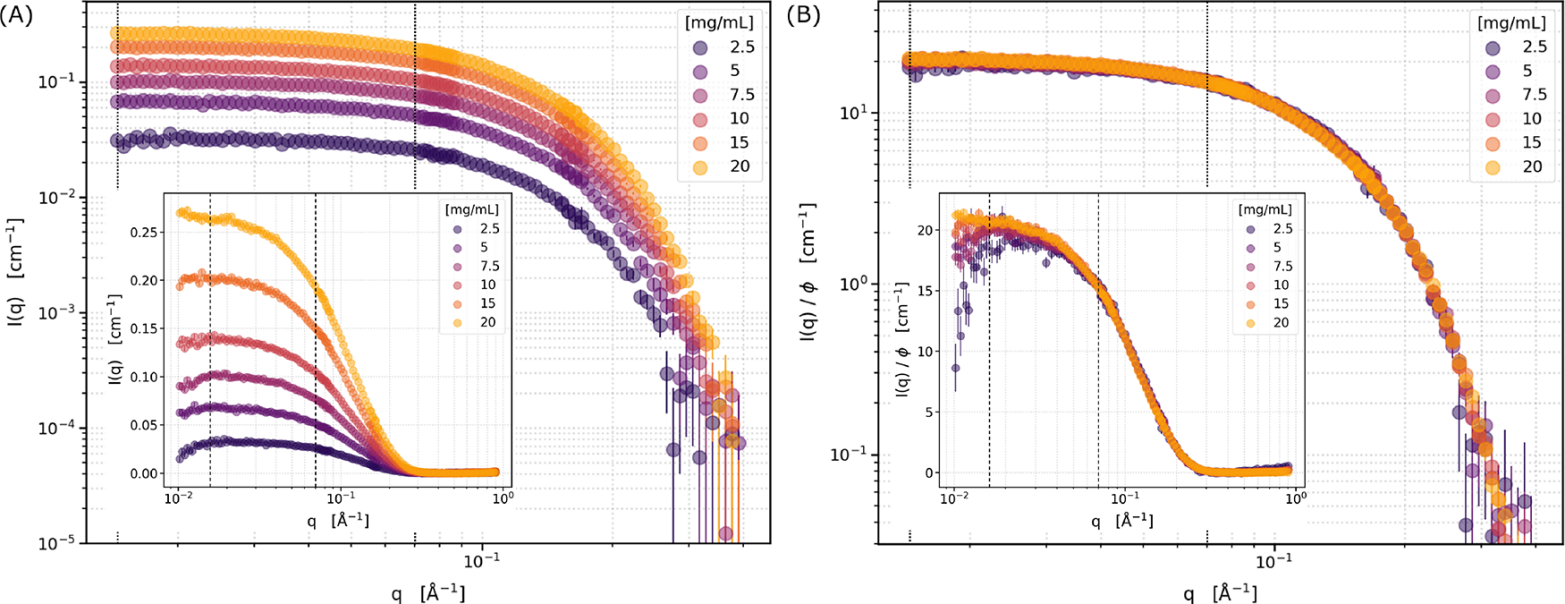
SANS data from solutions of lysozyme at 25 mmol/L histidine and 150 mmol/L NaCl in D_2_O, pH 6, at various nominal concentrations at 25°C. (A) After background subtraction in log–log scale. (B) Data normalized by the lysozyme volume fraction. Insets show data in lin-log scale. Dotted black lines indicate the *Q*-range used for subsequent Guinier analysis. Error bars indicate detector counting statistics as $1/\sqrt{N}$ , where *N* is the number of counts at a given *Q* bin during the exposure.

The modified Guinier analysis (eqs 9 and 15) was conducted over the range indicated by the black dotted lines in Figure 1, 0.015 Å < *Q* < 0.07 Å, using a custom Python code. As nominal lysozyme concentration increases from 2.5 to 20 mg/mL, *R*_*g*,obs_ *Q*_max_ ranges from 0.88 to 1.01 and the fit value of *R*_*g*,obs_ increases from 12.57 ± 0.34 Å to 14.41 ± 0.06 Å. Exact fit values for *R*_*g*,obs_, *I*(0), and associated errors are provided in Table S2. Linear fits to the data in ln­[*I*(*Q*)] versus *Q* ^2^ used to obtain these values are shown in Figure S2. The results are summarized in Figure 2. Note that the data at very low *Q* (shown in Figure 1 insets) were not used in the Guinier fits. The exact *Q*-range was not found to significantly affect the results of the Guinier analysis; variational analysis is provided in Figure S3. Residual analysis of the Guinier fits over the reported *Q*-range indicates the absence of significant irreversible aggregation, radiation damage, or other artifacts in the data (Figure S4).

**Figure 2:**
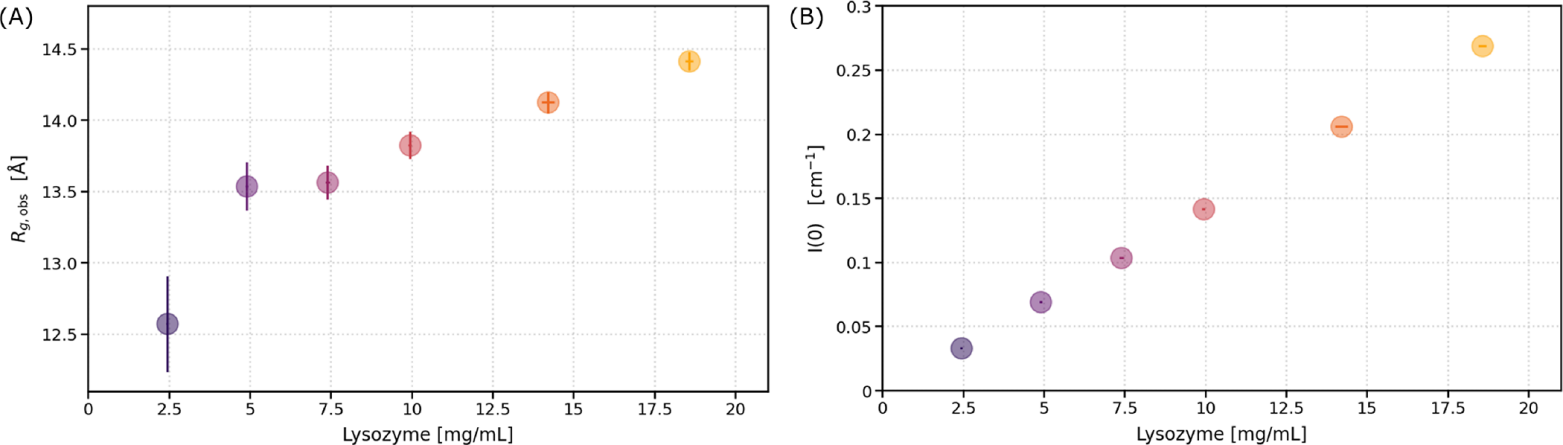
Results of the traditional Guinier fit to the low-*Q* lysozyme scattering intensity. (A) Radius of gyration. (B) Scattering intensity at *Q* = 0. Fits are shown for a representative *Q*-range of 0.015 Å < *Q* < 0.07 Å; variational analysis is provided in Figure S3. Error bars indicate the 1σ uncertainty of the least-squares Guinier fit value (*y*-error) and of the measured lysozyme concentration (*x*-error).

Our goal is to demonstrate the application of eq 9, in this case for relatively dilute solutions of lysozyme where eq 10 can be used to extract the relevant parameters. This requires replotting the *R*_*g*,obs_ values from the Guinier analysis (Figure 2) as *R*_*g*,obs_^2^ versus –*nB*_22_, where *B*_22_ is the second virial coefficient. Using the *I*(0) data from the Guinier fits, we fit a line to $\frac{I\left(0\right)}{n}$ versus *n*, where *n* is the lysozyme number density, as shown in Figure 3. We compute the second virial coefficient from this fit as $B_{22}=-\frac{a}{2b}$ , where *a* is the slope of the fit line and *b* is the *y*-intercept. For the example data shown, we obtain *B*_22_ = (−3.80 ± 0.56) × 10^–20^ mL. In other commonly reported units, this value is equivalent to *B*_22_ = –22900 ± 3400 mL/mol or *B*_22_ = (−1.12 ± 0.17) × 10^–4^ mol·mL/g^2^. Using this result, we can also calculate *S*(0) at each protein concentration via *S*(0,*n*) = 1 – 2*nB*_22_. The resulting *S*(0)-values, shown in Figure S5, indicate a deviation of 5% or less from *S*(*Q*) = 1 for all lysozyme solutions tested herein. In general, *S*(*Q*) slightly exceeds 1, which along with the small negative value of *B*_22_ is consistent with weak attractive interactions between particles in solution.

**Figure 3:**
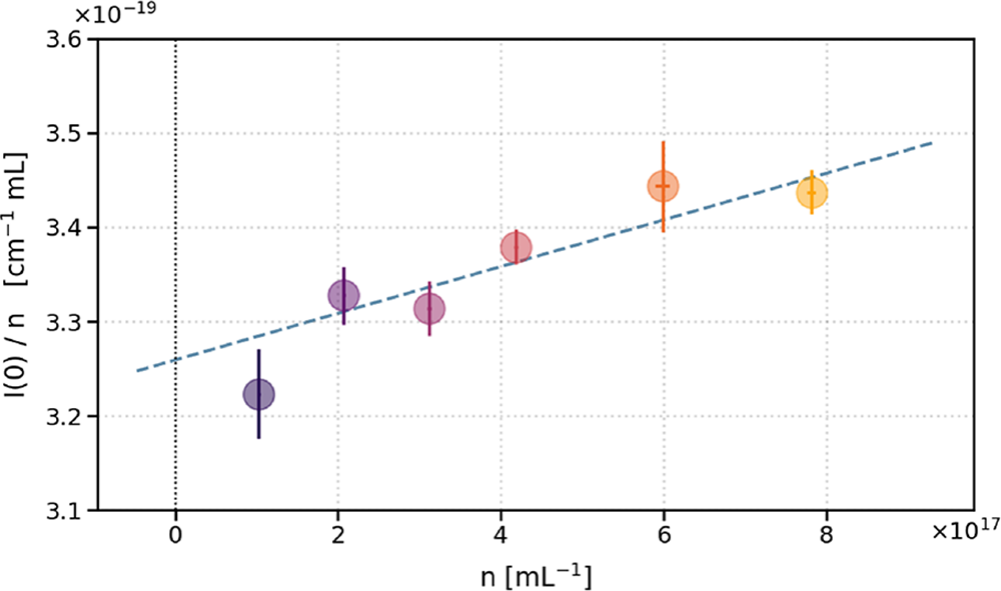
Obtaining the second virial coefficient (*B*_22_). A linear fit to *I*(0)/*n* data gives *B*_22_ [mL] as *B*_22_ = –*a*/2*b*, where *n* is the lysozyme number density, *a* is the slope, and *b* is the *y*-intercept.

Finally, we use *R*_*g*,obs_ and *B*_22_ to extract the radius of interparticle interaction, *R*_*i*_, and the infinitely dilute radius of gyration, *R*_*g*,0_, based on eq 14. Figure 4 shows a plot of *R*_*g*,obs_^2^ versus –*nB*_22_ since ξ becomes –*nB*_22_ at dilute conditions, along with the resulting linear fit which yields *R*_*i*_^2^ as the slope and *R*_*g*,0_^2^ as the *y*-intercept. We find that *R*_*i*_^2^ = 1431 ± 158 Å^2^, from which we obtain *R*_*i*_ = 38 ± 2 Å. In addition, we obtain *R*_*g*,0_^2^ = 168 ± 3 Å^2^, corresponding to *R*_*g*,0_ = 13.0 ± 0.1 Å. Thus, for this example data set of lysozyme protein in histidine buffer at pH 6 with added NaCl, we find that *R*_*i*_:*R*_*g*,0_ ≈ 2.9:1, as compared to the theoretical relationship for hard spheres for which *R*_*i*,HS_ = 2*R*_*g*,HS_ (see Section 2.4).

**Figure 4:**
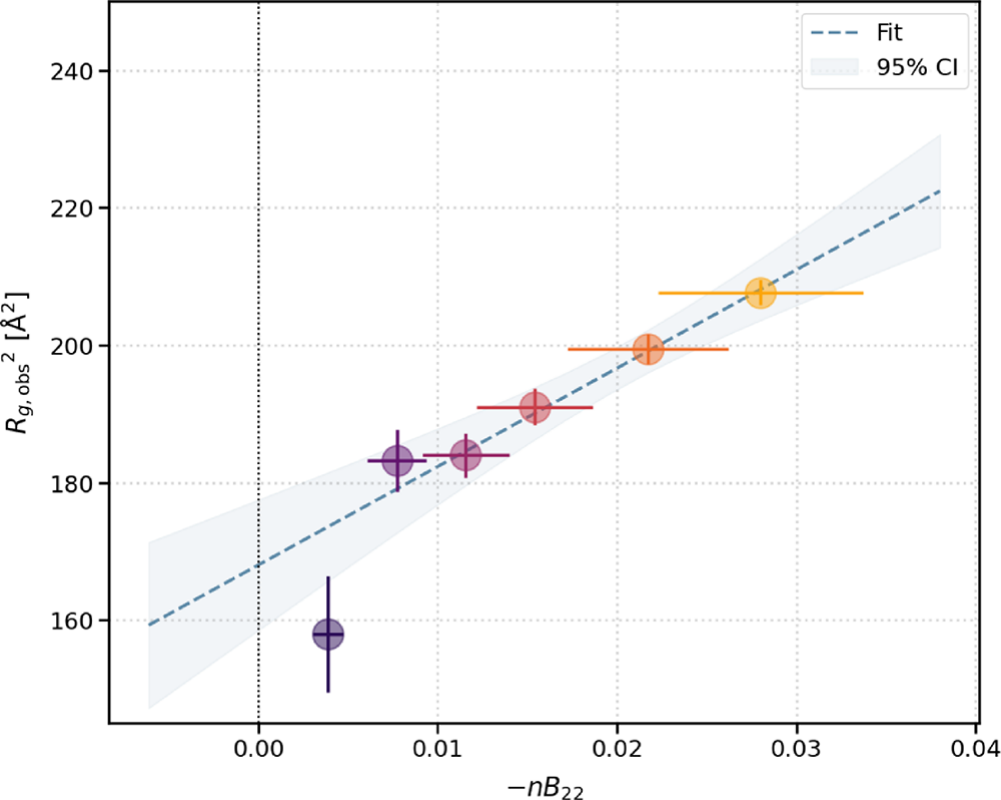
*R*_*i*_, the radius of interparticle interaction, and *R*_*g*,0_, the infinitely-dilute radius of gyration, are obtained from our modified Guinier analysis using this plot. The values of *R*_*g*,obs_ from the classical Guinier fit, along with the zero-angle structure factor *S*(0) as $\xi =\frac{1}{2}\frac{S\left(0\right)-1}{S\left(0\right)}$ , or ξ = –*nB*_22_ in the dilute case with *B*_22_ is the zero-angle second virial coefficient as shown here (*x*-axis), are used to create this plot. The linear fit (dotted blue line) returns the values of *R*_*i*_^2^ and *R*_*g*,0_^2^ as the slope and *y*-intercept, respectively. The shaded region indicates the 95% confidence interval of the fit line.

## 5 Discussion

Above, we showed that the “true” or infinitely dilute *R*_*g*_-value (*R*_*g*,0_) and the radius of interparticle interaction (*R*_*i*_) can be obtained from the low-*Q* asymptotic behavior of *I*(*Q*). We demonstrated this method using example SANS data on solutions of lysozyme, a small and well-studied prolate protein which is often used as a model protein for small-angle scattering measurements. As demonstrated here, the method is straightforward to apply for systems with relatively weak or intermediate interparticle interaction. Whether *R*_*i*_ is measurable for strong interactions will depend on the capability of a scattering instrument and how strongly the sample scatters. For strongly interacting samples, the concentration range for linearity in *R*_*g*,obs_^2^ is likely quite small, requiring good measurement accuracy over a range of low-concentration measurements. In addition, the method is straightforward and most accurately applied in the case of monodisperse solutes like proteins, but will need modification to obtain *R*_*i*_ for samples with significant polydispersity. For high concentration measurements, note that *R*_*i*_ works for *S*(*Q*) at low *Q* for any concentration and shape of particles and does not require a virial expansion, since it is related to the potential of mean force, ω­(**r**), which includes all correlations (see eq 11).

The quality of scattering data is very important to obtaining an accurate result for *R*_*i*_ and *R*_*g*,0_. In particular, due to the relatively weak scattering signal for dilute solution measurements, correct background subtraction is extremely important. To eliminate differences in background due to thickness variation between sample holder cell walls, we recommend either using the same cell to conduct buffer and sample measurements as in the data set reported herein, or the use of a flow cell. Still, the forward scattering intensity for the nominal 2.5 mg/mL sample shows a slight downturn likely due to low protein concentration and short data collection time, which may have resulted in fitting signal near the beamstop and account for its deviation from linearity in Figure 4. Therefore, depending on what concentrations correspond to minimal interparticle interaction for a given sample, future measurements of this type could easily push beyond measurable limits of current neutron scattering instruments, and require careful planning and quality evaluation prior to analysis.

Though the lysozyme proteins have attractive interactions, evidenced by the negative *B*_22_-value, interactions between them are weak so clusters that form as a result of the attractive interactions should be reversible.^34–36^ Thus, the residuals of the Guinier fit are qualitatively flat and randomly distributed about zero (Figure S3), rather than showing a characteristic “smile” shape that typically indicates the presence of irreversible aggregates. Our *B*_22_-value near −1 × 10^–4^ mol·mL/g^2^ is close to literature values in similar buffers and temperatures. At 50 mmol/L sodium acetate and pH 4.5 with 200 mmol/L NaCl in H_2_O, the lysozyme *B*_22_ at 20°C is −0.39 × 10^–4^ mol·mL/g^2^.^37^ Lysozyme at pH 6 in aqueous NaCl solution with total electrolyte concentrations 0.1 and 0.3 M in minimal citrate buffer have *B*_22_-values between −1 × 10^–4^ and −2.5 × 10^–4^ mol·mL/g^2^ at 25°C.^23^ Additionally, prior studies of lysozyme solubility in various buffer conditions similar to those used herein—namely, 150 mmol/L sodium chloride in 20 mmol/L histidine buffer, pH 6 in D_2_O at 25°C—have found the proteins are fully soluble in pure H_2_O at similar concentrations of sodium chloride and protein at slightly lower pH.^38–40^

The *R*_*g*_ of lysozyme has been extensively investigated before, and our results herein are consistent with the literature. A recent multinational round-robin study investigating protein size from small-angle scattering found values ranging from *R*_*g*_ = 12.16 ± 0.42 Å to *R*_*g*_ = 13.6 ± 0.33 Å for lysozyme in D_2_O depending on the measurement and calculation method. Batch SANS measurements of hen egg lysozyme protein (150 mmol/L sodium chloride in 50 mmol/L sodium citrate buffer, pH 4.5 in D_2_O) resulted in *R*_*g*,obs_-values in the range of 13–14 Å in Guinier analysis (excluding an outlier above 15 Å), with an average of 13.60 ± 0.33 Å where the error indicates one standard deviation. The averaged round-robin data were collected at a variety of concentrations in the dilute regime at the discretion of the participating beamline scientists; the study did not extrapolate the reported values to infinite dilution.^41^ Despite the different buffer, the reported round-robin average is equivalent within error to our *R*_*g*,obs_ average of 13.67 ± 0.10 Å.

As expected, both averaged Guinier *R*_*g*,obs_ values slightly exceed our extrapolated infinitely-dilute *R*_*g*,0_ value of 13.0 ± 0.1 Å. As shown in eq 14, for relatively dilute concentrations, the negative *B*_22_(0), orattraction between particles, results in a slight increase of *R*_*g*,obs_ with increasing concentration. On the other hand, positive *B*_22_(0), orrepulsion between particles results in a decrease in *R*_*g*,obs_ as the concentration increases. In both cases, *R*_*i*_ reflects the model-independent net displacement from ideality due to interparticle interaction.

*R*_*g*,0_ is relevant to all the tested lysozyme concentrations, in contrast to the values from Guinier analysis which change with concentration even though these are dilute solutions (i.e., minimal interparticle interactions). Our work highlights that the small deviations from *S*(*Q*) = 1 at low-*Q*, which reflect the interparticle interactions in our lysozyme solutions, do play a role in the observed *R*_*g*,obs_ value. This is true even in the dilute regime: *S*(*Q*) deviates above 1 by less than 6% in the tested concentration range (Figure S3). Our method enables a means to quantify the degree of this deviation from ideality by introducing the parameter *R*_*i*_.

However, the modified Guinier analysis method described herein does not necessarily require a dilute solution. In fact, the derivation of eqs 9 and 37, which describe *S*(*Q*) at low *Q*, only requires the assumption that *Q* → 0 without imposing any constraints on the concentration regime. Further, Guinier analysis requires the use of a dilute solution only because of the assumption that *S*(*Q*) = 1. We use dilute solutions for our example data because we expect that regime is likely to be most relevant to applications of the method, due to the direct relation between *R*_*i*_ and *V*(*r*) at low concentration (eq 13).

Since this work introduces the parameter *R*_*i*_ for the first time, no literature values are available to compare either the value of *R*_*i*_ (38 ± 2 Å) or its relative size compared to *R*_*g*,0_ found herein. As a point of reference, in Section 2.4 we calculated the ratio $\frac{R_{i}}{R_{g,0}}$ = 2 for the hard sphere potential, as compared to a value of 2.9 from our experimental lysozyme data. Given that lysozyme is a patchy and prolate solute in a liquid solution, we did not expect an agreement with the hard sphere result, since the hard sphere potential assumes perfectly spherical particles with only contact interactions. Yet, the experimental value of the ratio is still of similar order and magnitude. Since the low-*Q* scattering data only reflect the particle size, and some interaction information through *R*_*i*_, distinguishing spherical and anisotropic particles requires small-angle scattering data beyond the low-*Q* region, which one could obtain in a model-free way from *B*_22_(*Q*) data.^22^ More broadly, a deeper understanding of the magnitude of *R*_*i*_ and its relationship to *R*_*g*,0_ will require substantial further investigation beyond the scope of this study.

We anticipate that future efforts using the modified Guinier analysis method introduced herein will help establish the typical *R*_*i*_ values across a range of model proteins, polymers, and other colloidal systems of industrial relevance; their relation to *B*_22_ and *R*_*g*,0_; and comparison to simulations data. These endeavors will enable fundamental, model-independent insights about the length scale of the effective interaction potential in these experimental systems for the first time. Moreover, because *R*_*i*_ directly reflects the interparticle interaction potential between colloidal particles, it therefore may be relevant to predicting solution properties that are critical to a wide variety of industrial formulations.

## 6 Conclusions

In analogy to the Guinier radius of gyration (*R*_*g*_), we introduce a new parameter, the radius of interparticle interaction (*R*_*i*_), which is defined as the mean square displacement (MSD) of the total correlation function *h*(*r*). In dilute solutions, *R*_*i*_ is directly the MSD of the interparticle potential *V*(*r*). Herein, we present a model-independent extension to traditional Guinier analysis that enables simultaneous determination of both *R*_*i*_ and the “true” or infinitely dilute *R*_*g*_ value (*R*_*g*,0_) from the low-*Q* asymptotic behavior of *I*(*Q*). We derive a relationship between *R*_*i*_^2^, *R*_*g*,0_^2^, and the observed radius of gyration obtained by traditional Guinier analysis, *R*_*g*,obs_^2^, that holds for both spherical and anisotropic solutes. We also derive the modified or revised Zimm equation that incorporates interaction effects. We demonstrate the application of our modified Guinier analysis method using example SANS data from lysozyme solutions. We find that *R*_*g*,0_ = 13.0 Å, consistent with the reported literature values for lysozyme *R*_*g*_ at finite concentration in similar solvents. For *R*_*g*,obs_, the magnitude of the deviation from *R*_*g*,0_ is governed by *R*_*i*_, which is 38 Å for our example data set, indicating the length scale where interactions are significant in our dilute lysozyme solutions. Here, $\frac{R_{i}}{R_{g,0}}=$ 2.9, around 50% larger than the calculated value of 2 for the hard sphere potential. The method introduced herein both improves the accuracy in measurement and reporting of *R*_*g*_-values and provides a model-independent parameter *R*_*i*_ that directly reflects colloidal interaction potentials in solution. Therefore, we expect that determining the typical magnitude of *R*_*i*_ across a variety of colloidal systems of various identities, flexibilities, and interaction types and understanding its relation to macroscopic properties like high-concentration viscosity will prove useful to future fundamental and industrial research efforts.

## Supplementary Material



## Notes

Zimm derivation; SANS background subtraction; Guinier fits, variational analysis, and residuals; and zero-*Q* structure factors (PDF)
