# Supplementary material for: Low‑Q Asymptotic Behavior of the Effective Structure Factor Yields Model-Independent Radius of Interparticle Interaction (R i )
Source: ACS Meas Sci Au. 2025 Nov 7;6(1):35–45. doi: 10.1021/acsmeasuresciau.5c00099 (PMC12921611; doi:10.1021/acsmeasuresciau.5c00099)
Supplement: Supplementary file 1 [file tg5c00099_si_001.pdf]

Electronic Supporting Information for: "Low-Q Asymptotic Behavior of the Effective Structure Factor Yields Model-Independent Radius of Interparticle Interaction (R_i)"

Chelsea E. R. Edwards, Wellington C. Leite, and Yun Liu*
*yun.liu@nist.gov

October 9, 2025

Contents

1 Classical Zimm Equations **1**

2 SANS Data **2**

3 Guinier Analysis **3**

4 Structure Factor at $Q = 0$ **5**

1 Classical Zimm Equations

The relationship between our equation and the traditional Zimm equation is shown as follows. which are equivalent for small QR_g. Equation 39 in the main text is equivalent to

$$\frac{Kn}{I(Q)} \approx \Big(\frac{1}{M} + 2nA_{22}\Big)\Big(1 + \tfrac{1}{3}Q^2{R_g}^2\Big)\,, \tag{1}$$

where M is the molecular mass of a molecule, $A_{22} = \frac{B_{22}}{M}$, and $K = \frac{P(0)}{M}$. Distributing terms gives

$$\frac{Kn}{I(Q)} \approx \frac{1}{M}\Big(1 + \tfrac{1}{3}Q^2{R_g}^2\Big) + 2nA_{22} + \tfrac{2}{3}nA_{22}Q^2{R_g}^2\,. \tag{2}$$

As $Q^2 {R_g}^2$, is small in the Guinier region, the third term is small and can be neglected. Thus, Equation 2 can be rewritten in the more commonly-used form of the Zimm equation:

$$\frac{Kn}{I(Q)} \approx \frac{1}{M}\left(1 + \tfrac{1}{3}Q^2 {R_g}^2\right) + 2nA_{22}\,. \tag{3}$$

2 SANS Data

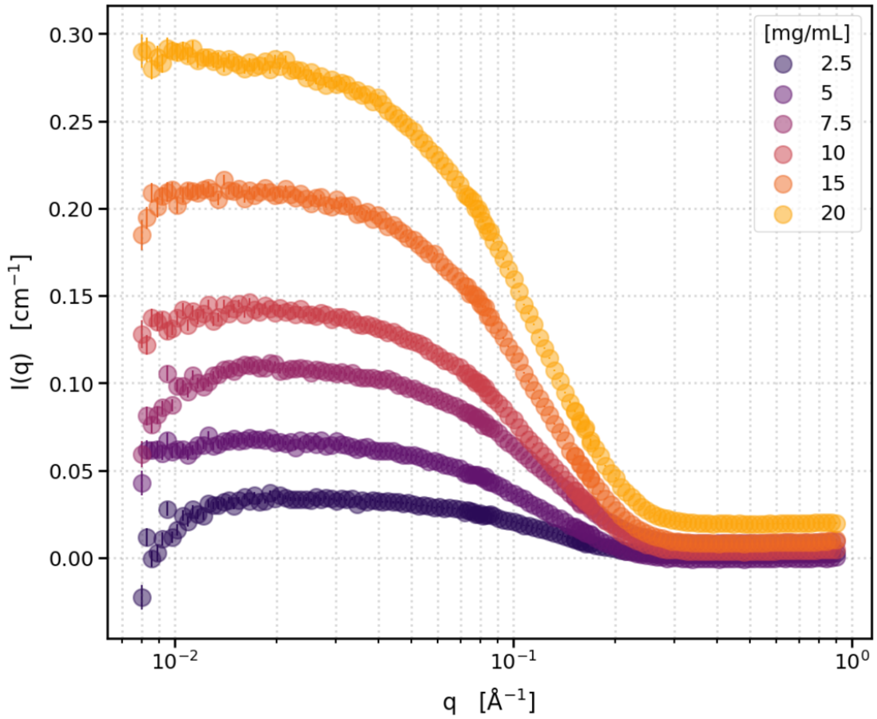

Figure S1: Buffer-subtracted SANS data from lysozyme solutions in 25 mmol/L Histidine (pH 6) and 150 mmol/L NaCl in D_2O at 25°C at various concentrations, prior to background correction.

Table S1: Background intensity values (I_B) used to correct the above SANS data through background subtraction, calculated by averaging the buffer-subtracted intensity between 0.35–0.45 Å at each concentration.

Nominal Concentration [mg/mL]	2.5	5	7.5	10	15	20
Mean I_B [cm^{-1}]	0.001646	-0.000626	0.008240	0.003620	0.008289	0.019557
Standard Deviation of I_B [cm^{-1}]	0.000023	0.000039	0.000032	0.000037	0.000054	0.000068

3 Guinier Analysis

Table S2: Guinier fit results from $I(q)$ data. Radius of gyration (R_g), forward scattering intensity (I_0), and the dimensionless product $R_g q_{\max}$ are shown for each protein concentration. Uncertainties are reported as standard deviations. The use of 3 decimal places here is indicated only to report the fit values actually used to generate the main-text plots and subsequent R_i fits, not to indicate that the measurement precision is that high.

**Nominal Concentration [mg/mL]**	**2.5**	**5**	**7.5**	**10**	**15**	**20**
$R_{g,\text{obs}}$ [Å]	12.571	13.535	13.564	13.821	14.123	14.411
Std. Dev. [Å]	0.336	0.167	0.118	0.096	0.075	0.064
I_0 [cm^{-1}]	0.0332	0.0688	0.1033	0.1415	0.2061	0.2687
Std. Dev. [cm^{-1}]	0.0002370	0.0002609	0.0002768	0.0003154	0.0003648	0.0004143
$R_{g,\text{obs}}Q_{\max}$	0.88	0.95	0.95	0.97	0.99	1.01

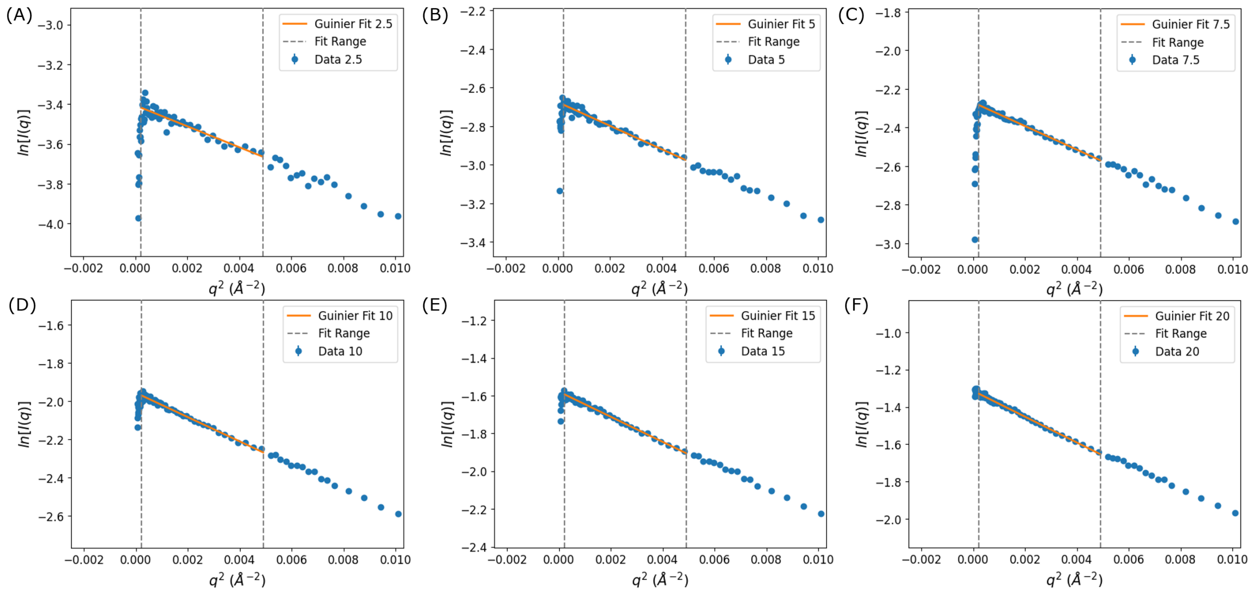

Figure S2: Guinier fits of the scattering data (blue dots) in $ln[I(Q)]$ versus Q^2 are shown at increasing nominal concentrations of (A) 2.5 mg/mL through (F) 20 mg/mL. Linear fits (orange) are shown, with the dotted black lines indicating the Q-range of each fit.

When averaging the R_g and I_0-values returned from the variational analysis below (Figure S3) for each nominal concentration, we obtain a value of the second virial coefficient as $B_{22} = -3.89 \times 10^{-20} \pm 8.18 \times 10^{-21}$ [mL]. This value differs from the B_{22}-value from the Q-range reported in the main text by about 11% of their uncertainty; in other words, their difference is insignificant given their error margins. Similarly, by using the average values from the variational analysis, the resulting value of ${R_{g,0}}^2$ is 167 ± 1 Å^2 and ${R_i}^2 = 1435 \pm 35$ Å^2. When accounting for

error, both values are equivalent to (not significantly different from) those reported in the main text.

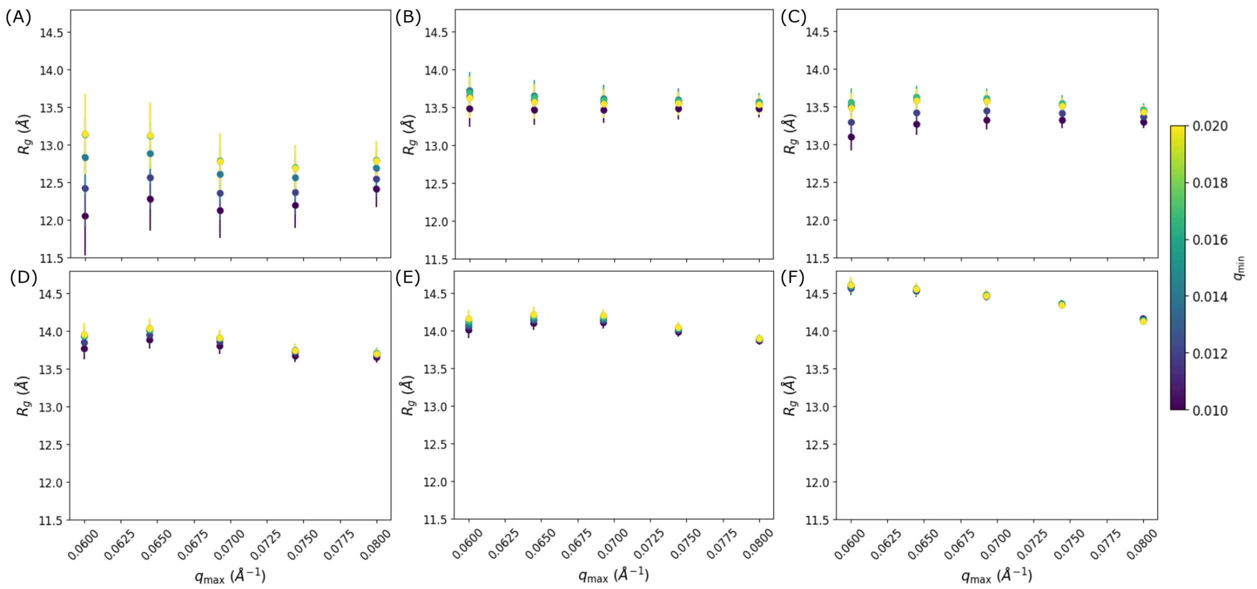

Figure S3: Guinier fits of the scattering data are shown for various fit ranges, with varying $Q_{\max}$ indicated in color and varying $Q_{\max}$ on the x-axis. Increasing nominal concentrations of lysozyme are shown as (A) 2.5 mg/mL through (F) 20 mg/mL.

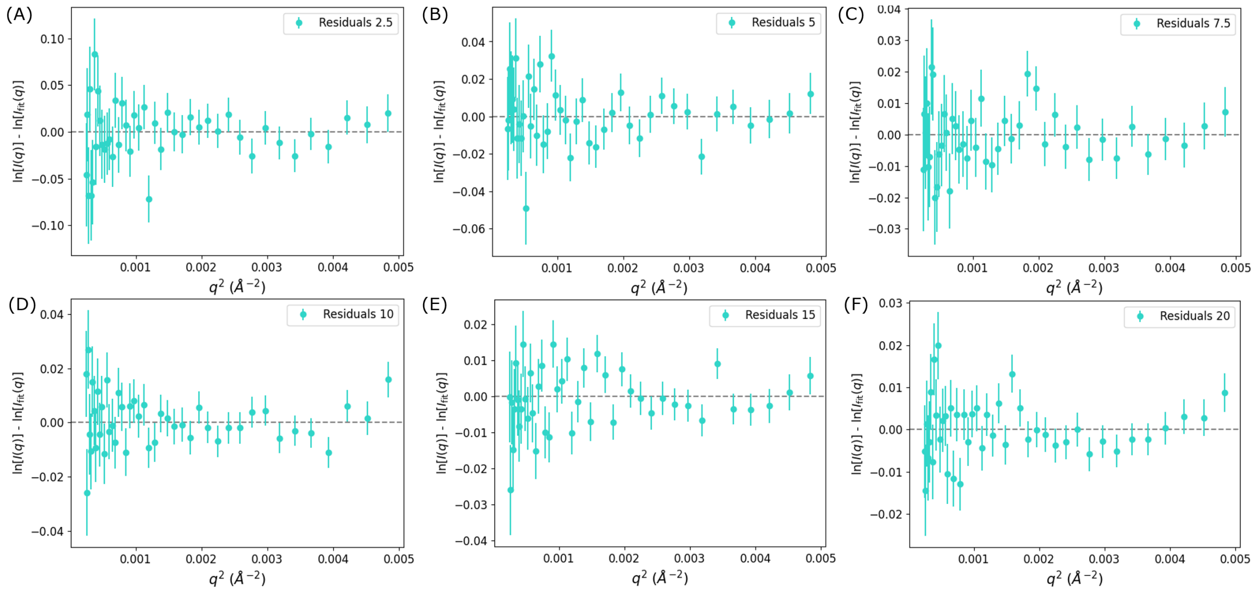

Figure S4: Over the Q-fit range used in Figure S2 and the main text, the residual is flat and randomly distributed about zero for all nominal concentrations.

4 Structure Factor at $Q = 0$

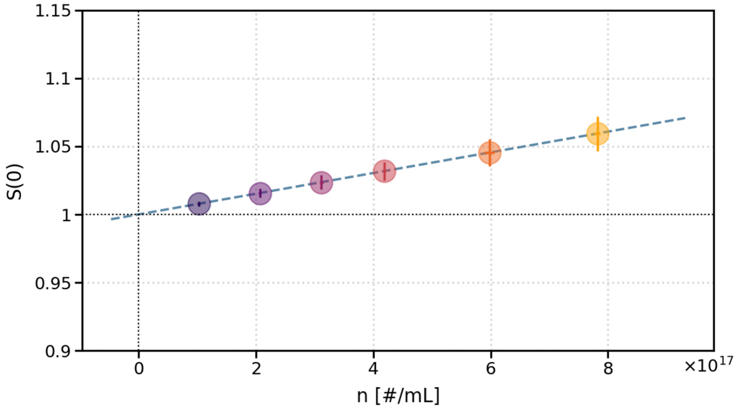

Figure S5: $S(0)$ is obtained at each n-value using $S(0,n) = 1 - 2nB_{22}$. The linear fit and extrapolation (dotted blue-gray line) illustrates that $S(0) \to 1$ at the infinitely dilute limit.
